# Cliniques

**Published:** 1860-05

**Authors:** N. S. Davis


### CLINIQUE BY PROF. N. S. DAVIS IN THE MEDICAL DEPARTMENT OF LIND UNIVERSITY.

Saturday Afternoon, April 4th, 1860.

Case 1st. *Impetigo of the Face.*—This is a child, aged about 5 years, light complexion, and delicate features, though not emaciated.

She complains of no sickness, but has an unpleasant eruption over the face, which you see existing in patches; one in front of the left ear, one on the left eye-brow, and one on the nose, with some isolated sores in the intermediate spaces. All the patches are covered with thick light yellow scabs, which have evidently been formed by the drying of matter, and the edges of which over-lap the sound skin, and somewhat obscure the original character of the eruption. These characteristics, however, are easily recognized in many of the isolated sores between the patches. Each of these are seen to consist of a slightly elevated base, surrounded by an areolar redness, in the centre of which is a flattened pustule, filled with pus.—They are thus seen to consist of regular pustules of impetigo. This form of eruption generally attacks children, who have been debilitated by previous disease, or with whom good food, clothing and cleanliness have been more or less neglected. In many cases each pustule passes through its successive stages to complete development in five or six days, becomes covered with a dry scab, which in three or four days more falls off, leaving a pale red cicatrix. In other cases, connected with more decided constitutional derangement, the pustules after coming to maturity, instead of cicatrizing, remain as superficial ulcers, the matter from which constantly drying up, increases the size and thickness of the scabs, until they present the large, thick, light brown color, as seen in the present case.

*Treatment.*—Many cases of this form of eruption, need no other treatment than a proper regulation of diet, and attention to cleanliness. When the disease has been more protracted, and the pustules continue to appear in successive crops, with but little disposition to cicatrize, some degree of medication becomes necessary. What this shall be depends entirely upon the condition of each individual patient; there being no *specific* remedies for this form of cutaneous disease. In the case here present, we find the tongue coated, the bowels inactive, and the appearance of a moderately scrofulous disposition. We shall consequently direct for her a powder of Pulv. Rhei 3 grs., and Hydrarg. Cum Creta 2 grs., to be given every night for three successive nights; after which she may take 15 gtts. each of Fluid Ext. of Cinchonæ and of Rheubarb, before breakfast and dinner each day. The object being to produce such a state of the secretions as will expel from the system all foreign and effete material, and to restore a vigorous and healthy action of the digestive organs. Locally, no applications should be made except such as are emollient or directly sedative, unless the scabs fall off, leaving superficial ulcers without any disposition to granulate. In the latter condition, the application once a day, of the solution of Sulphate of Copper, or Sulphate of Iron, of the strength of 4 or 5 grs. to the ounce of water, will often do good. In no case should any of the various stimulating unguents be applied to pustules of impetigo. We have often seen the disease greatly aggravated by the application of Sulphur, either dry or mixed with Cerate. Indeed, it is often produced by the too free use of this article for the cure of Scabies.

Case 2. *Apathæ and Bronchitis.* This is a child 18 months old; fleshy, but with a pale and anxious expression of countenance. The mother says it has had a severe, harsh cough, with some fever, during the whole of the past week. The respiration is short, and accompanied by a coarse mucous ronchus. The fever is much increased every night, and is nearly absent during the morning hours. The pulse is quick, and the tongue covered with a whiteish fur. The mucous membrane of the mouth is more red than natural, and contains several inflamed and ulcerated follicles. It is evident from the harshness of the cough, the mucous ronchus, and the shortness of breath, that the child has a sub-acute grade of bronchial inflammation. And a similar grade of inflammation extends to the mucous membrane of the fauces and mouth. The paleness of the lips and features, with the distinctly paroxysmal character of the fever, shows the addition of another influence which must not be overlooked. During the last two or three weeks, cases of this kind have been of frequent occurrence in this city; so much so as to constitute a moderate epidemic. The attacks among young children have been much the most numerous, though no age has been exempt.

It differs from ordinary simple bronchitis or caterrhal inflammation of the respiratory passages, in the fact that the fever accompanying the cases is almost uniformly remittent in its form, and often bears no strict relation either in its continuance or severity to the grade of the local inflammation.

Gastric and intestinal irritations are also common accompaniments, giving rise to vomiting, diarrhœa, and sometimes to well-marked dysenteric discharges. Owing to these conditions, the ordinary anodyne expectorants so generally resorted to with success in simple catarrhal bronchitis, have almost uniformly failed to relieve these cases, unless used in conjunction with anti-periodics and alteratives. Hence, we shall prescribe for this child as follows, viz:

| | | |
|---|---|---|
| ℞ | Sulph. Quinine, | 4 grs. |
| | Pulv. Doveri, | 4 grs. |
| | Hydrarg. Cum Creta; | 4 grs. |
| | Sacchar. Alba, | 20 grs. |

Mix and divide into four powders, one of which should be given every six hours. Also,

| | | |
|---|---|---|
| ℞ | Fluid Ext. Asclepias Tub. | ℥ j. |
| | Syrup of Ipecac, | ℥ j. |
| | Tinct. Opii et Camph. | ℥ j. |

Mix and give 30 gtts. every six hours, alternated with the powders.

After the four powders are taken, we will continue for one day longer the Quinine and Dover's Powder without the Hydrarg. Cum Creta. For the apthæ in the mouth we will direct a solution of Chlorate of Potassa, one drachm to three ounces of water, to be used as a gargle.

Case 3. *Asthma.*—This patient is a laboring man, native of Ireland, aged about 55 years. He says he has been afflicted nearly all the past winter with a severe cough, and so much difficulty of breathing during the night as to prevent his taking a recumbent position, and to deprive him of refreshing sleep. You can observe that his face has a slightly bloated aspect; expression sad, and the respiration constantly labored and wheezing. In addition to the constant dyspnœa, he has paroxysms of very great difficulty, when he feels such a sense of suffocation as to be very distressing; and the blood becomes so imperfectly decarbonized that the lips are livid, the nails leaden color, and the whole countenance bloated and dingy. At the same time the skin becomes cool, relaxed, and covered with perspiration; the pulse small and compressible, and the mind dull.

These paroxysms last from one or two hours to a whole night, and constitute what is commonly called Asthma or Phthisic. But Asthma is no more a disease than dropsy; but merely a symptom depending on some preceding and accompanying pathological condition. This pathological condition may be an organic disease of the heart, by which the circulation of blood through the lungs is disturbed; or it may be a simple morbid condition of the nervous filaments distributed upon the bronchial tubes, by which temporary spasmodic contractions are induced; or it may be a certain grade of inflammation in the bronchial mucous membrane. Hence, when a case of asthma comes before us, the first inquiry must be, what is the special pathological condition on which the paroxysms of dyspnœa depend? This can be answered properly only by a careful examination both in regard to the general symptoms and physical signs. In this patient neither auscultation nor percussion indicate any disease of the heart; but by extending the same kind of examination to the whole chest, we find over the anterior part of both sides a loud, harsh, inspiratory murmur, with a very prolonged wheezing expiratory sound; but neither dulness on percussion, nor broncophony.

Each member of the class in auscultating the chest of this patient, cannot fail to receive the impression that the bronchial mucous membrane is rougher and dryer than natural, and that the air escapes from the lungs with difficulty, as though escaping through tubes both contracted and dry. All this shows the existence of extensive bronchial inflammation, of that peculiar chronic grade which causes a morbid sensitiveness of the surface, with sufficient thickening of the membrane to narrow the calibre of the tubes, and diminished secretion. The absence of dulness over any part of the chest, proves the pulmonary tissue to be free from unnatural density, and consequently free from either tubercles or pneumonia. The indications for treatment in this case are, to promote an increased secretion from the mucous surface, and to allay the morbid sensitiveness of the nervous filaments connected with the inflamed membrane. The first, by relieving the fulness of the capillaries in the mucous membrane, would lessen the obstruction and the dryness, while the second would prevent those paroxysms of spasmodic action which constitute the distressing periods of dyspnœa. To accomplish both these objects, we shall direct this patient the following:

℞ Tart. Ant. et Pot., 2 grs.
Pulv. Opii, 10 grs.
White Sugar, ℈ii.

Mix and divide into eight powders. Give one powder each morning, noon, tea-time, and bed-time. If after these are all taken the bowels are constipated, we shall give a blue pill, followed by castor oil, sufficient to operate as a mild cathartic. Then give a mixture of Fluid Extract of Lettuce, ℥ii, and Fluid Extract of Lobelia ℥i, in doses of a tea spoonful four times a day. Numerous other combinations might be selected from the numerous expectorants and anodynes embraced in the materia medica, which would be calculated to accomplish the same objects, but we have not time to particularize them at present. The patient should avoid stimulating drinks, and rich or highly seasoned articles of food; and should wear flannel next to the skin. At this clinique, there was presented to the class one case of Tubercular Phthisis; one of intermittent, and another of remittent fever; but our space will not admit a report of these at present.

### CLINICAL REPORTS.

Mercy Hospital. Service of Dr. N. S. Davis. Tuesday, April 24th, 1860.

### MALE WARD, No. 1.

Case 1. *Broncho-Pneumonia.*—This case was brought to the notice of the class, and fully examined on Thursday last, which was the next day after his admission to the Hospital.

You will remember that his expression of countenance was then anxious and depressed; his breathing labored, with a dry wheezing ronchus; his pulse about 90 per minute and firm; tongue slightly coated with a white fur; and he complained of some nausea, with pain in the cardiac region; a sense of constriction across the chest, and a harsh severe cough, with very little expectoration. You will recollect that on examining him with the stethescope, you found the respiratory murmur much exaggerated, with a prolonged dry ronchus in expiration over both sides of the chest, except in the mammary and axillary regions of the left side, in which there was strongly marked sub-crepitant rale. The patient then told us that he had been sick with cough, difficulty of breathing, and pain in his chest for two or three months.

We then expressed the opinion that the patient was laboring under a Chronic Bronchitis, by which the bronchial mucous membrane had become thickened, its secretion diminished; and on which had supervened a pneumonic congestion of the middle and lower lobes of the left lung. To relieve these pathological conditions, we then directed for the patient a powder composed of Pulv. Opii 1½ grs., Tart. Ant. et Pot. ⅛ gr., and Hydrarg. Chlorid. Mit. 1 gr., to be given every three hours. He continued these two days, during which time his cough became less severe; expectoration more free; and the pain and tightness in the chest much diminished. The powders were then discontinued, and the following mixture given in their stead:

℞ Comp. Honey of Squills, Senega and Ant. ℥ i.

Tincture of Opium and Camphor, ℥ ii.

Tincture of Veratrum Viride, ʒ i.

Mix, and give a tea spoonful every four hours.

This he has continued until the present time. You now observe no difficulty of breathing while he is at rest, and on applying the stethescope to the chest, you find no prolonged dry wheezing ronchus on either side, and there is only a slight trace of the sub crepitant rale in the left mammary and axillary regions. The latter regions still show slight dulness on percussion, and the proper inspiratory murmur remains somewhat exaggerated over the infra-clavicular regions. He coughs but little, and expectorates a thick opaque mucus. It is thus seen that the condition of the pulmonary organs is greatly improved.

On further inquiry we find the patient has frequent pains in the abdomen ; a desire to pass urine oftener than natural, with some scalding; and the intestinal discharges, though not more than one or two in the twenty-four hours, yet are watery and unhealthy. The pulse remains a little accelerated and firm. These symptoms indicate a very general irritation of the mucous membranes throughout the system; and may account for the continuance of a *firm* pulse, while the general aspect of the patient is that of debility. The desire to urinate often, with scantiness of that secretion, also suggests the possibility of albuminuria, or Bright's disease of the kidneys.

The latter affection sometimes comes on very insidiously; producing mental despondency, indigestion, cardiac palpitation, and sometimes pulmonary congestions, with so little direct disturbance of the urinary organs, that neither the patient nor his physician suspects the true nature of the case. So true is this, that in all cases of protracted ill-health, or the frequent repetition of attacks of local symptoms without a manifest cause, the practitioner should not only make the usual general inquiries in regard to the urinary secretion, but should subject it to such chemical and microscopic tests as will determine positively its composition and qualities. Hence, we shall have some of this patient's urine saved to-morrow morning for examination, the results of which we will inform you at the next clinique.* At present we will omit the expectorant and sedative mixture which has been given during the last three days, and give something better calculated to allay the general irritation of the mucous membranes, and to promote a more free and diluted secretion from the kidneys. For these purposes we shall direct the following:

| | | | |
|---|---|---|---|
| ℞ | Pulv. Gum Benzoin, | | ʒ ii. |
| | Tinct. Opium, | | ʒ i. |
| | Pulv. G. Arabac, | aa. | ʒ iii. |
| | White Sugar, | | |

Rub together thoroughly, and add

* On examining the urine before the class the next clinic morning, it was found to contain both an excess of Phosphatic Salts, and a quantity of Albumen.

Syrup of Ipecac, ℥ i.
Mint Water, ℥ i.

Mix, and give a tea-spoonful every four hours. Also,

℞ Spts. Nit. Dulc. ℥ ii.
Tinct. Digitalis, ℥ ss.

Mix, and give a tea-spoonful every four hours, alternated with the emulsion. We direct the Benzoin in the emulsion for this patient instead of Oil of Turpentine, because it is less apt to produce nausea, and is almost as reliable in effects on certain morbid conditions of the mucous membranes.

The further progress of the case will be made known to you when you visit the Hospital again.

Case 2. *Sub-acute Rheumatic Inflammation of the Diaphragm.*—This man, aged about 30 years, a laborer, was admitted to the Hospital yesterday. He says he has been subject to pains in his right side for a few days at a time, for the last two years. Though generally located near the lower margin of the ribs, it sometimes changed to the shoulders, and sometimes to the left side. It has not usually been accompanied by fever or cough, and has seldom been sufficiently severe to prevent ordinary labor. Six or seven days since it commenced as usual in the right side, but in a day or two changed to the left, and became unusually severe. In that place it has continued until the present time. His pulse is now 85 per minute, moderately full; his tongue covered with a whitish fur; his skin dry, and slightly warmer than natural; his bowels inactive; his breathing short, with an inclination to dry cough, which is suppressed as much as possible on account of the great aggravation of pain which it induces. The pain at present is located in the region of the attachment of the diaphragm to the ribs from the left side of the spine to near the sternum, and is very greatly aggravated on attempting to take a full breath, or coughing, or making considerable movement of the body in any direction. The pain is also increased at night.

The severity of the pain, its increase by respiration and coughing, and its location in the side, would readily lead to the supposition that the patient had pleuritic inflammation.

While the fact that pains had long existed in the opposite side, changing occasionally to the shoulders and other parts; and that the present attack commenced in the right side; would rather indicate rheumatic inflammation of the diaphragm. Perhaps auscultation and percussion alone can enable us to form an exact differential diagnosis. If pleurisy existed in its first stage we should hear a friction sound on applying the ear or stethescope to the affected side; if in the second stage accompanied by effusion, we should have either a continuance of the friction or creaking, or decided dulness with absence of respiratory sound, according to whether the effusion was plastic or serous.

On the other hand, if the case be simple rheumatic inflammation, we should find none of these physical signs, but the natural respiratory murmur and the natural resonance, down to the diaphragm. After making a physical exploration, the latter was found to be the case; and consequently that the patient was affected with sub-acute rheumatism in the left portion of the diaphragm. Believing this to be the true diagnosis, we shall prescribe for the patient this morning as follows:

℞ Vinum Calchici, ʒ i.

Tinct. Cimicifuga, Rac. ℥ ii.

Mix, and give a tea spoonful every four hours. And at bed-time give a powder containing Pulv. Opium 2 grs., Nit. Potassa 10 grs., and Calomel 1 gr. If the latter does not cause the patient to sleep in two hours, repeat the dose.

The object is to increase all the excretory functions; especially those of the skin and kidneys; and so far destroy the pain as to enable the patient to sleep during the night.

